# Akirin1 promotes myoblast differentiation by modulating multiple myoblast differentiation factors

**DOI:** 10.1042/BSR20182152

**Published:** 2019-03-01

**Authors:** Wenqiang Sun, Shenqiang Hu, Jiwei Hu, Jiamin Qiu, Shuang Yang, Bo Hu, Xiang Gan, Hehe Liu, Liang Li, Jiwen Wang

**Affiliations:** Farm Animal Genetic Resources Exploration and Innovation Key Laboratory of Sichuan Province, Sichuan Agricultural University, Ya’an 625014, Sichuan, P.R. China

**Keywords:** Akirin1, Duck, MRF4, MEF2B, Myoblast differentiation

## Abstract

Akirin1 is found to be involved in myoblast differentiation. However, the mechanism by which the Akirin1 gene regulates myoblast differentiation still remains unclear. In the present study, we found that ectopic expression of Akirin1 promoted myoblast differentiation by increasing the expression of myogenic regulatory factor (MRF) 4 (*MRF4*) and myocyte enhancer factor 2B (*MEF2B*) mRNA. Additionally, we showed that ectopic Akirin1 induced cell cycle arrest by up-regulating *p21* mRNA. To further uncover the mechanism by which Akirin1 promotes myoblast differentiation, we showed that the enhanced Akirin1 increased the mRNA expression of P38α. Importantly, the enhanced MRF4 expression by Akirin1 can be abrogated by treatment of SB203580, a p38 inhibitor. Similarly, we found that enhanced MEF2B expression by Akirin1 can be abrogated by treatment with LY294002, a PI3K inhibitor. Together, our results indicate that Akirin1 promotes myoblast differentiation by acting on the p38 and PI3K pathways and subsequently inducing the expression of myoblast differentiation factors.

## Introduction

Myoblast differentiation is a multiple-step process. During this process, proliferating myoblasts permanently withdraw from the cell cycle and fuse to multinucleated myotubes and form myofibers [1]. Myoblast differentiation initiated by the expression of the MRFs (myogenic regulatory factors), such as Myf5, MyoD, MyoG, and MRF4 [2–4]. Myf5 and MyoD are mainly involved in muscle specification and trigger conversion of non-muscle cells [5,6], whereas MyoG and MRF4 act later during myogenesis and allow myotube formation and maturation [3,7]. This conversion ability is greatly enhanced when MRFs are co-transfected with MEF2s (myocyte enhancer factors 2s) (MEF2A–D) [8]. Members of these two families of transcription factors also synergistically activated reporter gene expression [8]. In addition to MRFs and MEF2s, cyclin-dependent kinase inhibitors, such as p21, are also essential for myogenic differentiation by regulating myoblast cycle arrest [9–11]. A tight regulation of differentiation is therefore critical for the production of functional muscle.

Akirin1 is a nuclear protein, which belongs to a family of highly conserved proteins called akirins [12]. Akirins have been identified as a cofactor that serve as an interface between a developmentally critical transcription factor and the chromatin remodeling machinery [13]. Previously, Akirin1 was found to be highly expressed in differentiating myoblasts [14], suggesting role of Akirin1 in regulating myoblast differentiation. Moreover, overexpression of Akirin1 in C2C12 cells results in early withdrawal of myoblasts from the cell cycle, enhanced and accelerated differentiation, and hypertrophy of myotubes. Most importantly, Akirin1 overexpression leads to increased and earlier expression of MyoD and increased secretion of another known differentiation inducing factor, insulin-like growth factor (IGF)-II (IGF-II). [14] However, how Akirin1 regulates myoblast differentiation remains to be elucidated.

In the current study, we sought to determine the role of Akirin1 in myoblast differentiation. We found that ectopic expression of Akirin1 promoted myoblast differentiation by increasing the expression of myoblast differentiation markers. Furthermore, the enhanced expression by Akirin1 can be abrogated by treatment of SB203580 or LY294002. Additionally, our findings showed that ectopic Akirin1 induces cell cycle arrest by up-regulating *p21* mRNA.

## Materials and methods

### Construction of the Akirin1 ectopic expression plasmid

According to the NCBI reference sequence of murine Akirin1 (GenBank accession no. NM_023423.3), the cDNAs encoding Akirin1 were amplified by PCR with P1 (Table 1). These cDNAs were cloned into the pMD19-T plasmid (TaKaRa, Japan) and named pMD19-T-Akirin1. The entire coding region of murine Akirin1 was digested from pMD19-T-Akirin1 plasmid and then inserted into the PEGFP-N1 plasmid via EcoRI and BamHI sites. After sequencing (Applied Invitrogen, China), the second recombinant plasmid was confirmed and named as pEGFP-N1-Akirin1. To evaluate its quality, the second recombinant plasmid was extracted and double-digested by EcoRI and BamHI (Beyotime Biotech, China).

**Table 1:** Primer sequences used in the present study

Primer ID	Primer name	Primer sequence (5′–3′)	Product size (bp)	Ta Opt (°C)
P1	Akirin1-1F	CGGAATTCGCCACCATGGCGTGCGGGGCGAC	576	58
	Akirin1-1R	CGGGATCCTCAGGATACATAGCTTGTTGGCCTTGTC		
P2	Akirin1-2F	ACTCAGCCTTCCTCCTCGGCACTTAC	261	60
	Akirin1-2R	TCAGGATACATAGCTTGTTGGCCTTGTC		
P3	MyoG-F	CGGATCACCTCCTGCCTGA	87	60
	MyoG-R	CGTCCTCTACGGCGATGCT		
P4	MRF4-F	GGAGCGCCATCAGCTACATC	132	56
	MRF4-R	CGAGGAAGTCCGAGCCATT		
P5	MEF2A-F	GGGTATGATGCCACCATTGAA	170	55
	MEF2A-R	GGTCTGCGCTAGTCAAGGAGTAA		
P6	MEF2B-F	CCACGCCATCAGCATCAAGTCAG	103	60
	MEF2B-R	GGCTTCGCTCAGGGAGGTCAG		
P7	MEF2C-F	ATGGACAAAGTGCTGCTGAAA	272	55
	MEF2C-R	TGGTTGGACACTGGGATAGAAA		
P8	MEF2D-F	GCGTCCTGTGCGACTGCGAGAT	156	58
	MEF2D-R	ACGTCTCGATGATGTCCGCGTTG		
P9	p21-F	TTGGCTCACAAGGTCCCATCTAAGG	161	56
	p21-R	TTGGCTCACAAGGTCCCATCTAAGG		
P10	P38α-F	ACTGGATACAGACAAGCGAATTACTG	99	54
	P38α-R	TAGGGGTCTGCCACTGGTTCA		
P11	P38β-F	TGATGATGAGATGACTGGATACG	136	55
	P38β-R	CTTCCCTTTCAATAGTTCTGCC		
P12	P38γ-F	GAAGATTCTGGATTTTGGATTGG	127	55
	P38γ-R	GATGTCAACTGTTTGCGTGTAATG		
P13	IGF-Ⅱ-F	CAGTGGGACGAAATAACAGGA	115	54
	IGF-Ⅱ-R	CGCTCAGACTTGACGGACTT		
P14	PI3K-F	CTTTTACCGAGGAGGTTCTGTGG	137	60
	PI3K-R	CTGAAGGTTGGTCTTTGTGGAC		
P15	Akt-F	TCTTTGCTGGCATTGTTTGGC	152	60
	Akt-R	GCTGTCATCTTGGTCAGGAGGAGT		
P16	GADPH-F	AAGGCTGAGAATGGGAAAC	254	60
	GADPH-R	TTCAGGGACTTGTCATACTTC		

Abbteviations: F, forward primer; R, reverse primer; Ta Opt, annealing temperature.

### Cell culture

For each experiment, Peking duck eggs which were incubated for 13 days were randomly selected, and the duck myoblasts used were isolated from the leg muscles of embryos [15]. Cells were cultured in growth medium (GM) containing Dulbecco’s modified Eagle’s medium (DMEM) (Tokyo, Japan), 15% FBS (Invitrogen, U.S.A.), and antibiotics (100 U/ml penicillin and 100 g/ml streptomycin). Until approximately 80% confluence was reached, the medium was switched to differentiation medium (DM) which contained DMEM and 2% horse serum (Invitrogen, U.S.A.). The present study was carried out in strict accordance with the recommendations in the Guide for Sichuan Agricultural University Animal Care and Use Committee, Sichuan Agricultural University, Sichuan, China.

### Ectopic expression and drug treatments

For myoblast proliferation experiments, growing myoblasts (70–80% confluence) were transfected with pEGFP-N1-Akirin1 or the pEGFP-N1 empty plasmids using Lipofectin3000 (Invitrogen, U.S.A.) according to the manufacturer’s instructions. At 24 h post-transfection, cells were harvested for RNA and protein extraction. For myoblast differentiation experiments, pEGFP-N1-Akirin1 plasmids or the pEGFP-N1 plasmids were transfected for 24 h before the induction of differentiation. At 12, 24, 36, and 48 h after the induction of differentiation, cells were harvested for RNA and protein extraction, respectively. For LY294002 or SB203580 treatment experiments, pEGFP-N1-Akirin1 plasmids or the pEGFP-N1 plasmids were transfected for 24 h before the induction of differentiation, when GM was switched to DM, LY294002 (20 μM, PI3K inhibitor) (Beyotime Biotech, China) or SB203580 (10 μM, p38 MAPK inhibitor) (Beyotime Biotech, China) were added into the duck myoblasts [16]. After LY294002 or SB203580 treatment, cells were harvested for RNA and protein extraction, respectively.

### Real-time PCR

Total RNA was extracted from myoblasts by TRIzol (Takara, Japan) according to the manufacturer’s instructions and then measured by spectrophotometry. RNA was reverse-transcribed to synthesize the cDNA by using the reverse transcript system (Takara, Japan). Real-time PCR (RT-PCR) was carried out with the SYBR Prime Script RT-PCR Kit (TaKaRa, Japan) by using the Bio-Rad CFX Manager (Bio-Rad Laboratories, U.S.A.). Each sample collected from cells was repeated with three biological samples. The relative expression of target genes was normalized against the internal control gene, duck GAPDH. The relative gene expression was analyzed by the comparative *C*_t_ method (2^−ΔΔ*C*_t_^ method) [17], and primer sequences (P2-P16) were used for RT-PCR (Table 1). In order to determine the RT-PCR efficiency of the target and internal control genes, ten-fold serial dilutions (10^−1^–10^−5^) of cDNA were produced and assayed in triplicates to yield standard curves. The identity of the amplified products was also confirmed by sequencing (Applied Invitrogen, China).

### Western blot

Total cellular proteins were extracted from duck myoblasts with RIPA lysis buffer (Beyotime Biotech, China). Protein samples were resolved by 10% SDS/PAGE and electroblotted on PVDF membranes (Beyotime Biotech, China). The membranes were incubated in block buffer (Beyotime Biotech, China) at 37°C for 2 h and then incubated with the primary antibody at 4°C for 12 h. After that, the membranes were incubated with a secondary antibody at 37°C for 2 h, and subsequently detected by using the ECL kit (Beyotime Biotech, China) and a Gel Imaging System (Bio-Rad, U.S.A.). Primary antibodies against the following proteins were purchased from Biosynthesis Biotechnology and used at 1:1000 dilutions: p38α, p-p38α, MRF4, Akt, p-Akt, while GAPDH (diluted 1:1000) was purchased from Beyotime Biotech. The secondary antibodies (HRP–conjugated goat anti-rabbit IgG or goat anti-mouse IgG) were purchased from Biosynthesis Biotechnology as well. ImageJ software (National Institutes of Health, U.S.A.) was used for densitometry analysis. The values below each Western blot image represent the relative abundance of the target protein compared with GAPDH.

### Flow cytometry

After 24 h of transfection, the duck myoblasts were switched to DM for 12 h, and then these myoblasts were collected for cell cycle phase detection. The cell suspension was filtered and washed twice with 0.1 mol/l (pH 7.4) cold PBS. After that, the cells were resuspended in PBS at a concentration of 1 × 10^6^ cells/ml. Then, 1 ml of the suspension was transferred to a 5 ml culture tube and centrifuged at 200×***g*** for 5 min. The supernatant was discarded, and 1 ml of PI staining solution (5 μl/ml propidium iodide, 0.5% Triton X-100, 0.5% RNase, PBS) was added. The cells were gently whirled and incubated for 20 min in the dark at room temperature (25°C). Then, 2 ml of PBS were added and centrifugal elutriation was performed. The supernatant was discarded, and cells were resuspended in PBS, the cell phases were analyzed by flow cytometry (FACSCalibur, BD, Franklin Lakes, NJ, U.S.A.).

### Immunofluorescence staining

After 24 h of transfection, myoblasts were switched to DM for 48 h, and then these cells were used for immunofluorescence (IF) staining. The cells were harvested and fixed with 4% paraformaldehyde (PFA) for 20 min and treated with 0.2% Triton X-100 for 10 min at room temperature, then blocked with 5% normal goat serum in PBS for 1 h. After incubation with primary antibodies against MyHC (diluted 1:1000, DSHB) overnight at 4°C, the cells were incubated against FITC–conjugated secondary antibody (Biosynthesis Biotechnology, China). The slides were co-stained with DAPI (Beyotime Biotech, China) to visualize the nuclei. The relative areas of positive staining were evaluated with ImageJ software.

### Statistical analysis

The data were subjected to ANOVA and the means were compared for significance by Tukey’s test. ANOVA and *t* test were performed using SAS (SAS Institute, Cary, NC, U.S.A.) and the results were expressed as the mean ± S.D.

## Results

### Ectopic expression of Akirin1 increases duck MRFs transcription and myotube formation

**Figure 1:**
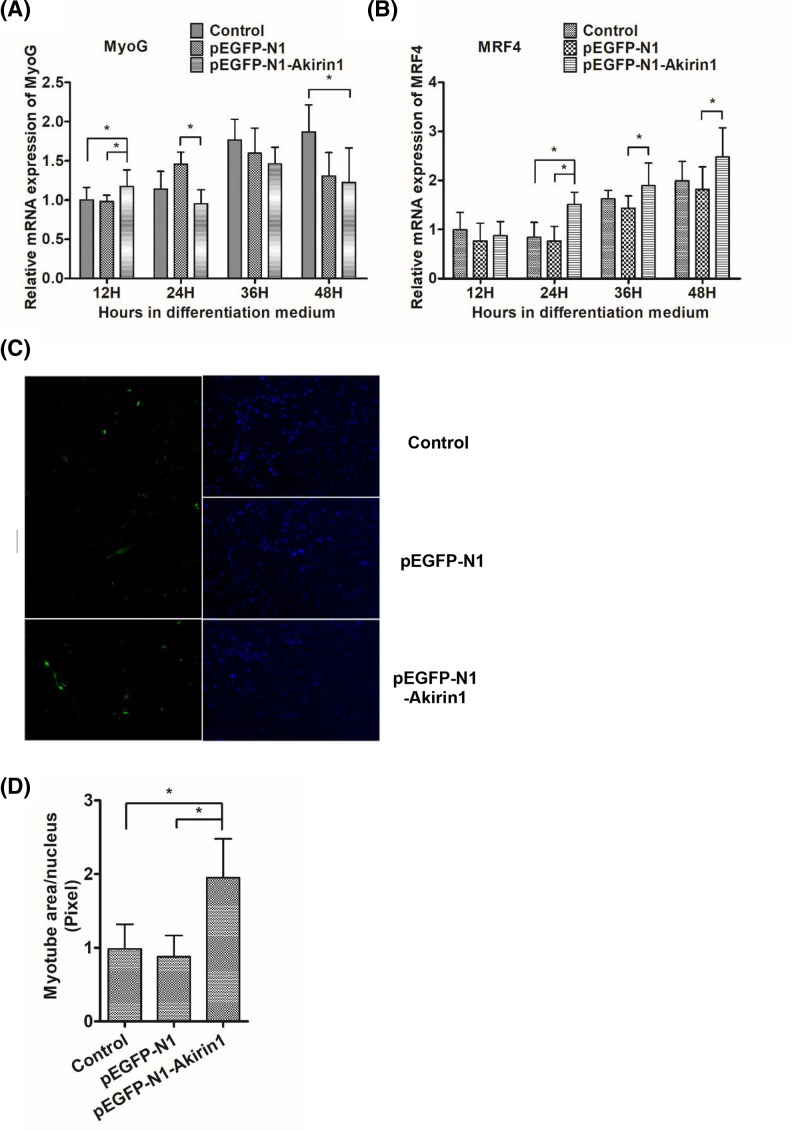
Influence of Akirin1 ectopic expression on *MRFs* mRNA expression and myobute formation After transfecting with duck pEGFP-N1-Akirin1 or pEGFP-N1 plasmids for 24 h, myoblast differentiation was induced by switching the cells to DM for 12–48 h. (**A,B**) The mRNA expression of MyoG and MRF4. (**C,D**) MyHC expression in myotubes detected by IF (right panel, green color), and quantitation of the positive myotubes area and normalized against the total number of nuclei (left panel, purple color). Each point represents the relative mean ± S.D. *, *P*<0.05.

To determine the role of Akirin1 in duck myoblast differentiation, primary duck myoblast was transiently transfected with a pEGFP-N1 vector or a pEGFP-N1 vector expressing Akirin1 from 12 to 48 h. We found that the level of MyoG was enhanced by Akirin1 at 12 h, but decreased after 24 h with no significance (Figure 1A). Additionally, we found that the level of MRF4 transcript was up-regulated by Akirin1 expression at 24 h (Figure 1B). Consistent with this, we found that that the number of MyHC-positive cells in the pEGFP-N1-Akirin1 group was more than in the two control groups (Figure 1C,D). Together, these data suggest that Akirin1 promotes differentiation in primary duck myoblasts by enhancing differentiation factors.

### Ectopic expression Akirin1 induces cell cycle arrest in primary duck myoblasts

**Figure 2:**
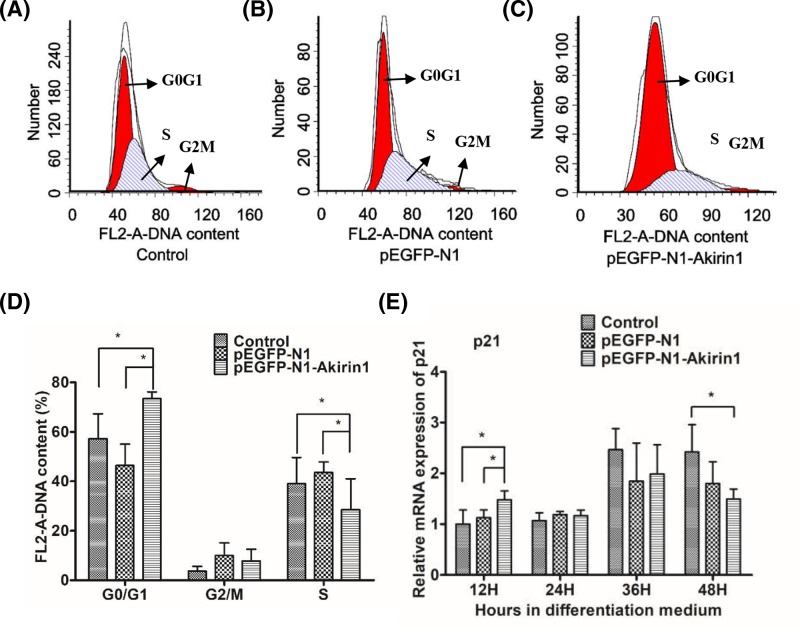
Influence of Akirin1 ectopic expression on cell cycle arrest (**A**–**D**) Cell cycle changes in duck myoblasts by flow cytometry (above panel) and digital conversion histogram (below panel). Propidium iodide staining for DNA content and an FACSCalibur flow cytometer were used to determine the percentage of cells in G_0_/G_1_, G_2_/M and S. (**E**) The mRNA expression of p21. Each point represents the relative mean ± S.D. *, *P*<0.05.

It is known that MRFs and cyclin-dependent kinases, such as p21, coregulate each other and regulate cell cycle arrest, which is essential for myogenic differentiation [18]. Thus, to examine whether Akirin1 induces cell cycle arrest, flow cytometry analysis was performed using primary duck myoblasts with or without Akirin1 expression. We found that, the percentage of cells arrested in G_0_/G_1_ phase was markedly increased in cells with ectopic expression of Akirin1 as compared with that in the two control cells (Figure 2 A–C). The relative percentage of cells in G_0_/G_1_, G_2_/M, and S phases was quantitated and shown in Figure 2D. Since p21 is known to be critical to induce G_1_ arrest, thus, we performed RT-PCR analysis and found that ectopic expression of Akirin1 significantly up-regulated p21 transcript at 12 h but not at later stage (Figure 2E).

### Effects of Akirin1 ectopic expression on the p38 MAPK signaling pathway

**Figure 3:**
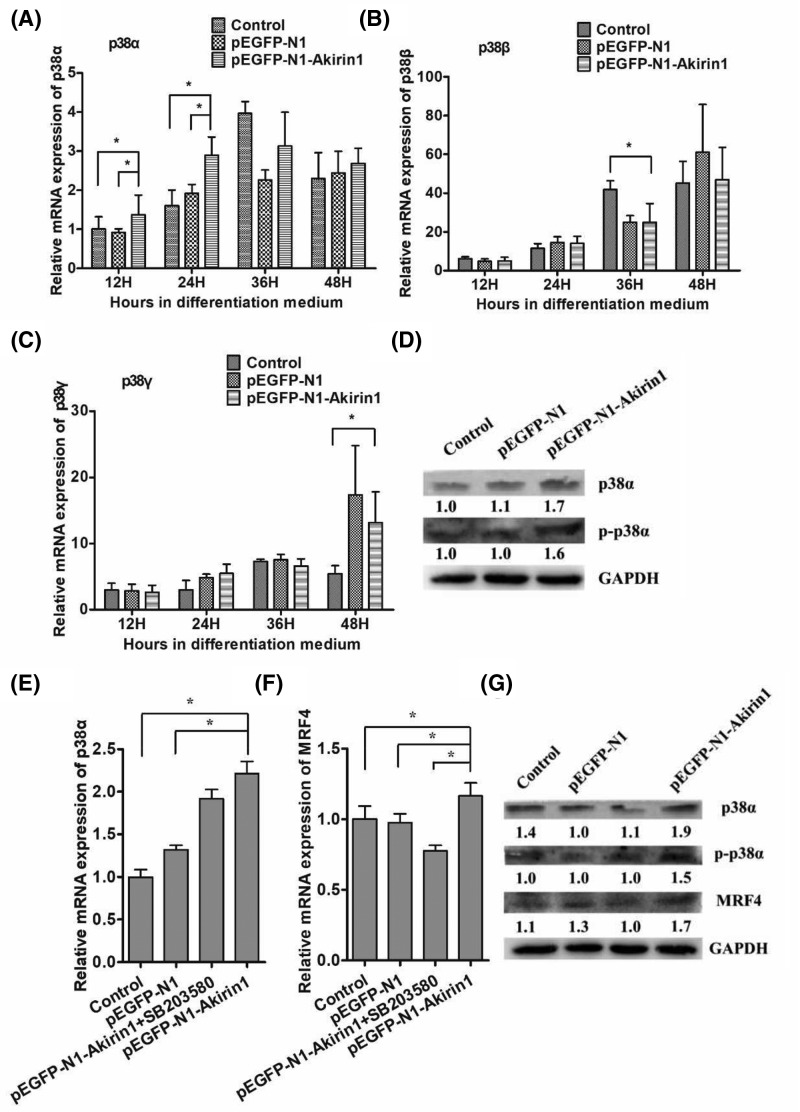
Effects of Akirin1 ectopic expression and SB203580 on p38 MAPK signaling pathway At 24 h post-transfection in the GM, RT-PCR and Western blot were carried out. (**A**–**D**) Influence of Akirin1 ectopic expression on *p38α, p38β*, and *p38γ* mRNA expression and p38α protein expression. (**E**–**G**) Effects of SB203580 and Akirin1 ectopic expression on the expression of p38α and MRF4. The values below each Western blot image represent the relative abundance of target protein compared with GAPDH. Each point represents the relative mean ± S.D. *, *P*<0.05.

Recent studies have demonstrated that p38 MAPK co-operates with the myogenic transcription factors in the activation of a subset of late-transcribed genes, hence contributing to the temporal expression of genes during differentiation [19]. Thus, to further determine the mechanism by which Akirin1 promotes duck myoblast differentiation, the level of several p38 isoforms including p38α, p38β, and p38γ was examined. We found that the level of p38α, but not p38β and p38γ was up-regulated by Akirin1 (Figure 3A–C). Furthermore, the protein expression of p-p38α also increased (Figure 3D). To verify this, the p38 inhibitor, SB203580, was used to treat primary duck myoblasts with or without Akirin1 expression and the level of MRF4 transcripts was determined. We showed that SB203580 was unable to abrogate the up-regulation of p38α transcript by Akirin1 (Figure 3E). Importantly, we showed that treatment with SB203580 abolished the up-regulation of MRF4 by Akirin1 (Figure 3F). Consistent with this, we found that the level of MRF4 protein was decreased by SB203580 (Figure 3G).

### Ectopic expression of Akirin1 up-regulates MEF2s transcript

**Figure 4:**
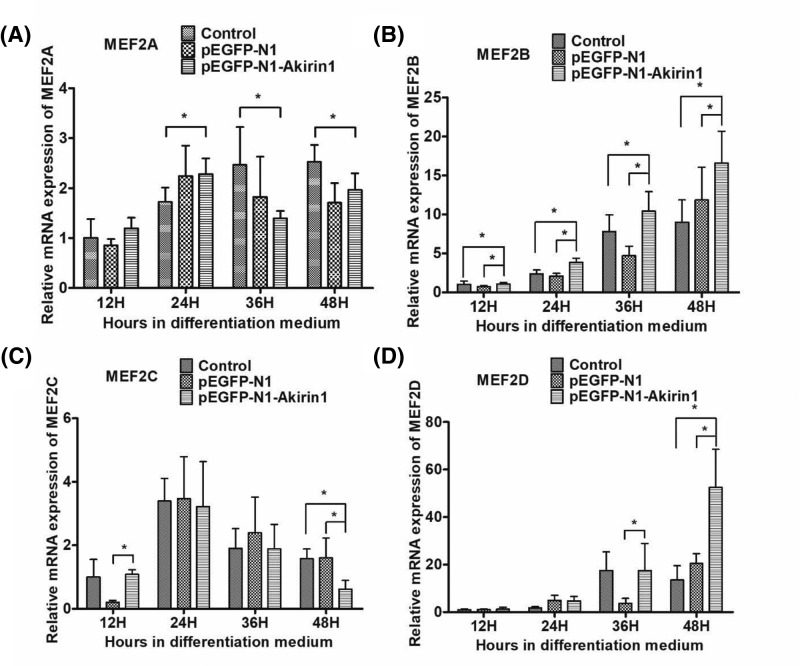
Influence of Akirin1 ectopic expression and LY294002 on *MEFs* mRNA expression (**A**) Influence of Akirin1 ectopic expression on *MEF2A* mRNA expression. (**B**) Influence of Akirin1 ectopic expression on *MEF2B* mRNA expression. (**C**) Influence of Akirin1 ectopic expression on *MEF2C* mRNA expression. (**D**) Influence of Akirin1 ectopic expression on *MEF2D* mRNA expression. After transfecting with duck pEGFP-N1-Akirin1 or pEGFP-N1 plasmids for 24 h, myoblast differentiation was induced by switching the cells to DM for 12–48 h. Each point represents the relative mean ± S.D. *, *P*<0.05.

MEF2s family transcription factors cannot induce myogenesis in transfected fibroblasts, but when coexpressed with the MRFs, they dramatically increase the extent of myogenic conversion above that seen with either MRF factors alone [20]. To examine the role of Akirin1 in regulating MEF2s, qRT-PCR analysis was performed. We showed that the expression of MEF2A and MEF2C irregularly increased to a high level and then decreased (Figure 4A,C), while the expression of MEF2B and MEF2D progressively increased to a high level (Figure 4B,D). Interestingly, the level of MEF2B was significantly up-regulated by Akirin1 in a time-dependent manner (Figure 4B) whereas the level of MEF2D was up-regulated at late stage (Figure 4D).

### Effects of LY294002 and Akirin1 ectopic expression on IGFs/PI3K/Akt/MEF2B signaling pathway

**Figure 5:**
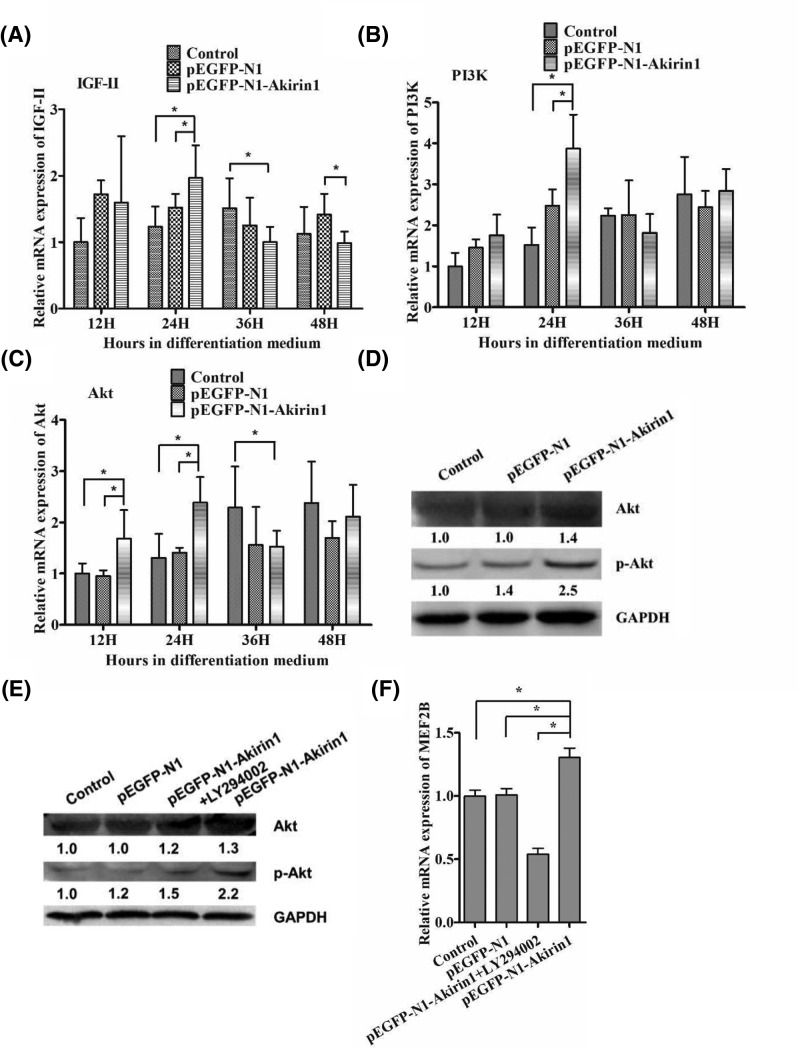
Effects of Akirin1 ectopic expression and LY294002 on PI3K/Akt signaling pathway (**A**–**D**) Ectopic expression of Akirin1 increased the expression of IGF-Ⅱ/PI3K/Akt signaling pathway related proteins. (**E,F**) Effects of LY294002 and Akirin1 Ectopic expression on the expression of Akt, p-Akt, and MEF2B. At 24 h post-transfection in the GM, RT-PCR and Western blot were carried out. The values below each Western blot image represent the relative abundance of target protein compared with GAPDH. Each point represents the relative mean ± S.D. *, *P*<0.05.

Previous studies showed that IGFs/PI3K/Akt signaling pathway plays an important role in myoblast differentiation [21]. To determine whether IGFs/PI3K/Akt pathway is involved in Akirin1-mediated up-regulation of MEF2s, the expression of IGF-II, PI3K, and Akt was examined. We showed that ectopic expression of Akirin1 significantly increased the mRNA expression of IGF-II, PI3K, and Akt at 12 and 24 h (Figure 5A–C). Furthermore, the expression of p-Akt Ser^473^ also increased (Figure 5D). To verify this, the PI3K inhibitor, LY294002, was used to treat primary duck myoblasts with or without Akirin1 expression. We showed that LY294002 was able to abrogate the up-regulation of p-Akt Ser^473^ protein by Akirin1 (Figure 5E). Importantly, we showed that treatment with LY294002 abolished the up-regulation of MEF2B by LY294002 (Figure 5F).

### Effects of LY294002 and Akirin1 ectopic expression on cell cycle arrest

**Figure 6:**
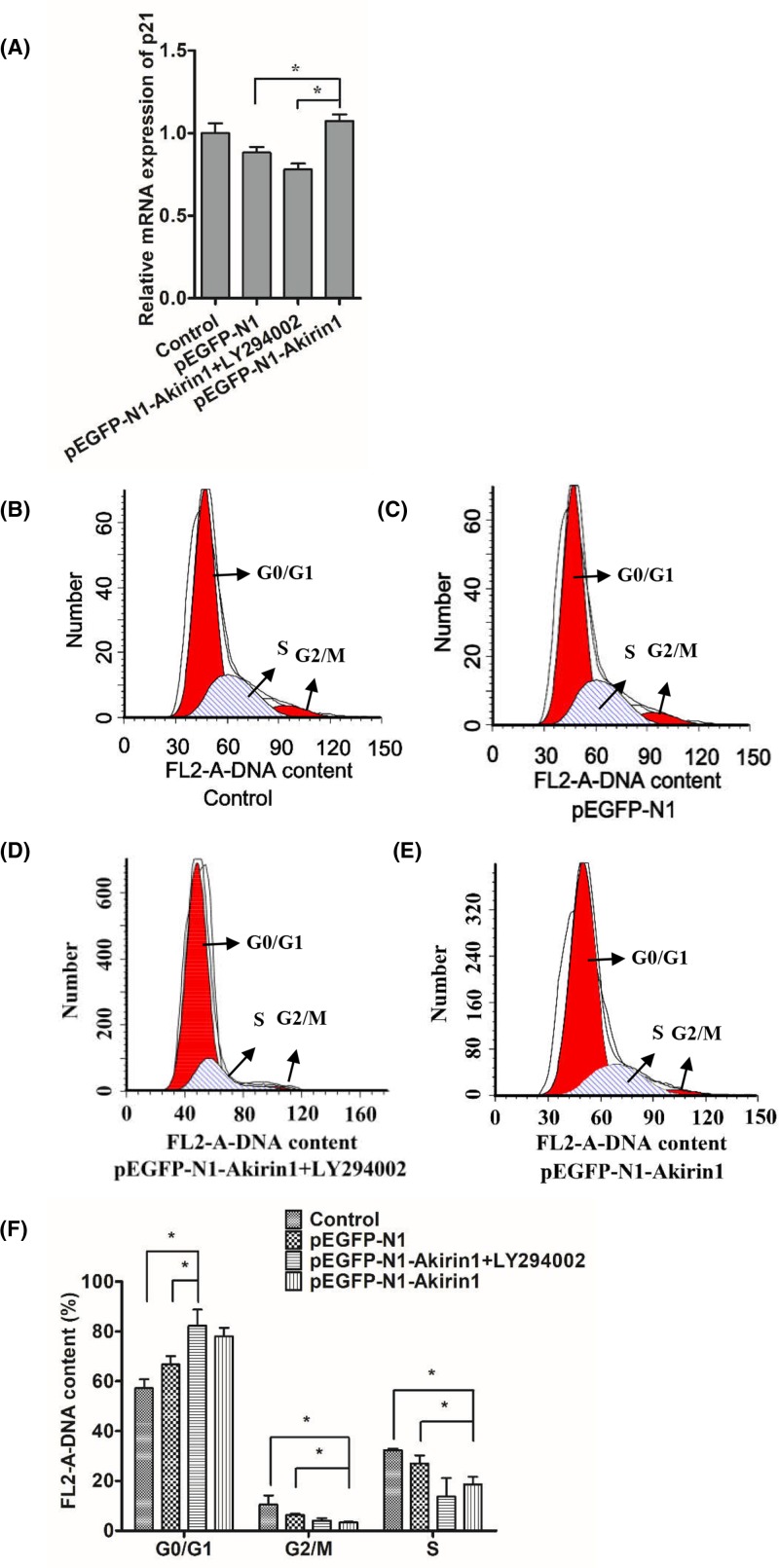
Effects of Akirin1 ectopic expression and LY294002 on cell cycle arrest (**A**) Effects of LY294002 and Akirin1 ectopic expression on the expression of p21. (**B**–**E**) Effects of LY294002 and Akirin1 ectopic expression on the cell cycle arrest by flow cytometry and digital conversion histogram (**F**). Propidium iodide staining for DNA content and an FACSCalibur flow cytometer were used to determine the percentage of cells in G_0_/G_1_, G_2_/M, and S.

To determine the mechanism by which Akirin1 regulates cell cycle arrest during myoblast differentiation, primary myoblasts transiently expressing with or without Akirin1 were treated with or without LY294002. We found that the increased p21 transcript by Akirin1 was abrogated upon treatment with LY94002 (Figure 6A). Next, we performed an FACS analysis. Interestingly, we found that treatment with LY294002 was unable to recover the cell cycle arrest by Akirin1 (Figure 6B–E). Quantitative data indicate that Akirin1 increased the number of cells arrest in G_1_ phase, which can be further increased by treatment of Akirin1 (Figure 6F).

## Discussion

Myoblast differentiation is a highly ordered process that is initially induced by several MRFs [22]. Previous studies showed the expression of MyoG, which is normally expressed at the onset of differentiation, were found to be increased by ectopic expression of Akirin1 [14]. In this study, we found that ectopic expression of Akirin1 not only significantly increased the mRNA expression of MyoG at 12 h, but also significantly increased the mRNA expression of MRF4 which plays a critical role in initiating myoblast differentiation as well [23]. In the terminal myoblasts differentiation, myoblasts gradually differentiate into multinucleated myotubes, meanwhile, MyHC coexpresses during this stage [22,24,25]. Previous studies demonstrated that overexpressed Akirin1 could increase the number of myotube [14]. In this study, ectopic expression of Akirin1 could also increase the number of MyHC-positive cells. Thus, this finding suggested that ectopic expression of Akirin1 could influence the myoblast differentiation by mediating the expression of several MRFs.

When the myoblast differentiation was initiated, myoblasts also began to exit from the cell cycle [10]. This process is controlled by cyclin-dependent kinase inhibitors, such as p21 [9]. A previous study described that overexpressed Akirin1 could increase the expression of p21 which suggested that Akirin1 may be able to accelerate cell cycle arrest [14]. In the present study, the number of cells at the G_0_/G_1_ stage in the pEGFP-N1-Akirin1 group was significantly higher than other two control groups, while the number of cells at the S stage in the pEGFP-N1-Akirin1 group was significantly lower than two control groups, which suggested that ectopic expression of Akirin1 significantly accelerated the cell cycle arrest. Moreover, the mRNA expression of p21 was significantly increased at 12 h after ectopic expression of Akirin1. Thus, these results suggested that Akirin1 could accelerate cell cycle arrest by some unknown ways, and then promote myoblast differentiation.

It is well known that IGFs including IGF-I and -II promote cell proliferation and differentiation through activation of the PI3K/Akt signaling pathway [21,26]. Interestingly, previous studies have shown that Akirin1 could increase the promoter activity of IGF-II which could act as an activator of the p38 MAPK signaling pathway [27]. p38 MAPK signaling pathway has been demonstrated that it plays an important role in stimulating the transcriptional activities of MRFs [28]. In this study, our result showed that ectopic expression of Akirin1 significantly increased the mRNA expression of p38α from 12 to 24 h, suggested that Akirin1 could promote the expression of p38α. Previous study found MRF4 could be phosphorylated by p-p38, resulting in its reduced transcriptional activity [29]. In this study, after Sb203580 treatment, the expression of MRF4 decreased to a low level suggesting that Sb203580 could block the positive impact of Akirin1 on the expression of MRF4 at the early stage of muscle differentiation. Thus, these results showed that Akirin1 could promote the duck myoblast differentiation through enhancing the mRNA expression of MRF4 mediated by p38 MAPK pathway.

It is well known that MRF and MEF2 family members co-operate in the activation of many muscle structural genes during differentiation and the formation of multinucleated myotubes. For example, MEF2 family members could regulate the expression of MRF4 by activating its promoter [30]. Moreover, MEF2B is capable to interact with the heterodimer that formed between MyoG and E12 to further promote the myoblasts differentiation [8]. In this study, our result showed that ectopic expression of Akirin1 could significantly alter the mRNA expression of MEF2B and MEF2D, suggesting that Akirin1 can regulate MEF2B and MEF2D expression. Previous study found that MEF2C is the first MEF2 gene to be expressed in the somite myotome (∼E9.0), while MEF2D expressed later than MEF2C [31]. Thus, Akirin1 may promote later myoblast differentiation via regulating the part of MEFs expression. Previous studies showed that the PI3K/Akt signaling pathway, which could be active by IGF-II, played an important role in myoblast differentiation [32–34]. In this study, ectopic expression of Akirin1 in the duck myoblast not only significantly increased the mRNA expression of IGF-II, but also significantly increased the expression of PI3K and Akt at 12 and 24 h. This finding suggested that Akirin1 could active the PI3K/Akt signaling pathway at the early stage of myoblast differentiation process. It has been demonstrated that p-Akt could increase the MEF2s transcriptional activity [35]. Interestingly, in the present study, after adding the LY294002, the mRNA expression of MEF2B significantly decreased to a lower level, suggested that LY294002 could also block the positive impact of Akirin1 on MEF2B. Thus, these findings suggest Akirin1 could promote the duck myoblast differentiation through enhancing the mRNA expression of MEF2B mediated by the PI3K/Akt signaling pathway.

It has been reported that Akt could phosphorylate p21 and then enhance its protein stability [36]. In this study, after adding the LY294002, the mRNA expression of p21 significantly decreased to a lower level, suggesting that p21 could be regulated by the PI3K/Akt signaling pathway. However, after adding the LY294002, the cell cycle arrest has not been changed, suggested that the cell cycle arrest was not regulated by PI3K/Akt signaling pathway. Thus, the more sophisticated regulatory mechanisms of Akirin1 on cell cycle arrest still need to be explored.

In conclusion, our results demonstrated that Akirin1 promotes myoblast differentiation by acting on the p38 and PI3K pathways and subsequently inducing the expression of myoblast differentiation factors. Additionally, Akirin1 could also promote cell cycle arrest in an unknown way. Further research is needed to clarify how Akirin1 promotes cell cycle arrest.

## Abbreviations

DM: differentiation medium
DMEM: Dulbecco’s modified Eagle’s medium
FBS: fetal bovine serum
GM: growth medium
IGF: insulin-like growth factor
MEF: myocyte enhancer factor
MRF: myogenic regulatory factor
MyHC: Myosin heavy chain
PVDF: polyvinylidene difluoride
RT-PCR: real-time PCR
